# A Comprehensive Review of *Moringa oleifera* Bioactive Compounds—Cytotoxicity Evaluation and Their Encapsulation

**DOI:** 10.3390/foods11233787

**Published:** 2022-11-24

**Authors:** Oana Lelia Pop, Andreea Diana Kerezsi, Călina Ciont (Nagy)

**Affiliations:** 1 Department of Food Science, University of Agricultural Sciences and Veterinary Medicine, 400372 Cluj-Napoca, Romania; 2 Molecular Nutrition and Proteomics Lab, CDS3, Life Science Institute, University of Agricultural Science and Veterinary Medicine, 400372 Cluj-Napoca, Romania; 3 Gembloux Agro-Bio Tech, Department of Food Science and Formulation, University of Liège, B-5030 Gembloux, Belgium

**Keywords:** *Moringa oleifera*, bioactive compounds, cytotoxicity, extraction techniques, encapsulation

## Abstract

*Moringa oleifera* Lam. has gained a lot of attention due to its potential use as a functional food not only for human health but also for animal health. Its bioactive molecules include carbohydrates, phenolic compounds, carotenoids, fatty acids, essential amino acids, and functional peptides. Despite significant efforts to isolate and characterize bioactive metabolites with health functions, few effective metabolites are accessible. The current review aims to describe the main processes for extracting and encapsulating bioactive compounds from *Moringa oleifera* for potential impact on food science and public health. Researchers have shown that different extraction techniques significantly impact the Moringa polysaccharides’ molecular structure and biological activity. Encapsulation has been proposed to reduce oxidative stability and entrap active agents within a carrier material to deliver bioactive molecules into foods. Currently, polysaccharides and proteins, followed by lipids, are used for material encapsulation. Recent techniques include spray drying, cross-linking gelation, freeze-drying, nanoencapsulation, electrospinning, and electrospraying. Moreover, these encapsulations can overlap concerns regarding the *Moringa oleifera* compounds’ cytotoxicity. Future studies should prioritize the effect of new encapsulation materials on Moringa extract and develop new techniques that consider both encapsulation cost and efficiency.

## 1. Introduction

*Moringa oleifera* Lam. is gaining worldwide appreciation, especially for its extensive nutritional profile and for combating the malnutrition problem. The plant is rich in phytochemicals (flavonoids, isothiocyanates, phenolic acids, tannins) and has many nutrients, such as proteins, fibers, minerals, and a balanced composition of amino acids. It includes 13 species of shrubs and trees native to India and Africa, and it is distributed in many other tropical and arid countries [1,2].

According to different studies, the bioactive components of Moringa plants are developed in novel functional foods or they have other applications in the cosmetic and pharmaceutical fields. *M. oleifera* leaf powder may act as a dietary supplement when it is added in different types of food, such as sauces or soups. Furthermore, scientists show that the nutritional and quality value of cereals, cookies, bread, yogurts, cakes, or cheese was improved after adding the Moringa leaf powder. Moringa is also rich in ben oil, considered a substitute for olive oil due to its similar characteristics, containing oleic acid, sterols, and tocopherols. The plant can be utilized in the food industry as a natural additive as well; thus, it might replace synthetic ones in the future [3,4,5,6,7].

Moreover, almost every part of the plant (seeds, leaves, flowers, bark, roots, fruit, and immature pods) can be exploited because it offers a multitude of human health benefits due to its antibacterial, antidiabetic, anti-inflammatory, antioxidant, and hepatoprotective properties [8]. Aside from the diversity of therapeutic properties, Moringa is also known for its anticancer activity. One of the most common diseases, cancer, affects many people, and one in seven dies because of inadequate medication. This is the reason why many medicinal plants are being investigated to help treat this problem, including *M. oleifera.* The leaves that contain benzyl isothiocyanate, glucosinolates, and niazimicin are believed to be responsible for its anticancer activity [9].

The encapsulation technique is applied in order to have better access to the bioactive compounds found in *Moringa oleifera*. This technique protects the compounds, and it can be an excellent physical barrier against different conditions such as temperature, water activity, pH, oxygen, and light conditions. Moreover, the stability is improved by encapsulation [10]. Prior to encapsulation, the bioactive compounds need to be extracted. Several techniques are used (i.e., supercritical fluid extraction, microwave-assisted extraction, and cold pressing) [11].

Different factors, such as solvent type, extraction temperature, time, pH, extraction methods, or solvent concentration, can impact the efficacy of bioactive compounds release. For example, 12 bioactive compounds in *Moringa oleifera* seeds were identified as having the optimum conditions for the extraction: ethanol extraction (49.8%) at 80 °C, 45 min extraction time, and 0.62 mm particle size [12]. Through the encapsulation method, several carriers or coatings are used to protect the bioactive compounds, such as biopolymers, plant-based or animal-based proteins, polysaccharides, and gums [13]. Cytotoxicity of the carrier materials has become an interesting topic for researchers. In a study conducted in 2020, the lack of toxicity of tragacanth gum used in spray drying of Moringa was confirmed by a scientific group. However, more studies in this direction must be addressed [14].

The research of the studies meant to be included in this review was performed in Web of Science, Google Scholar, and Pubmed using the following keywords: *Moringa oleifera*, alkaloids, antimicrobial, antioxidant, bioactive compounds, carotenoids, cytotoxicity, diabetes, emulsion, encapsulation, extraction, fatty acids, folates, glucosinolates, hepatoprotective, isothiocyanates, metabolic syndrome, obesity, oil, phenolic compounds, polyphenols, polysaccharides, proteins, peptides, saponins, spray drying and tannins. We included all the studies that brought relevant information and were not redundant.

In this way, our paper provides an overview of the main processes for extracting and encapsulating the bioactive compounds of *Moringa oleifera* and their cytotoxicity. Thus, several extraction techniques (solvent, hot water, solid-state fermentation, ultrasound, microwave) are described to perform the encapsulation of the bioactive profile of Moringa (microencapsulation, nanoencapsulation, electrospinning, and electrospraying). To our knowledge, this is the first article that unites two essential aspects, the cytotoxicity of the bioactive compounds and their encapsulation, and has an impact on food science and public health.

## 2. Bioactive Components of *Moringa oleifera*

It is well known that, due to the rapidly growing drug resistance, scientists are shifting their attention to finding alternatives for bioactive compounds, especially those found in medicinal plants [15]. Because of Moringa’s high resistance to arid conditions and the fact that almost every part of the tree is valuable, its application varies from medicine usage and functional food preparation to biodiesel production and water purification [16]. Among the most important bioactive compounds of *M. oleifera* are carotenoids, phenolic compounds, alkaloids, glucosinolates, isothiocyanates, folates, tannins, saponins, and fatty acids [1].

Flavonoids (apigenin, quercetin, luteolin, myricetin, kaempferol), lignans (secoisolariciresinol, isolariciresinol, medioresinol, epipinoresinol glycosides), and phenolcarboxylic acids and their derivatives (coumaroylquinic, caffeoylquinic, feruloylquinic acids) are the main phenolic compounds found in Moringa leaves. The hydroxyl groups are responsible for the antioxidant activity [1,17,18].

Carotenoids are natural pigments in plants and foods, and they are responsible for protecting against cellular damage by acting as antioxidants. In research conducted by Saini (2014), six main carotenoids were observed, which include 13-Z-lutein, all-E-lutein, all-E-luteoxanthin, all-E-zeaxanthin, all-E-β-carotene, and 15-Z-β-carotene [19].

Different secondary metabolites are found in *Moringa oleifera* leaves, such as alkaloids, glucosinolates, and isothiocyanates. Myrosinase, an enzyme found in the idioblasts of the cell plant, is activated when the plant is damaged, processed, harvested, or chewed and produced β-D-glucose hydrolysis when the pH was neutral, resulting in isothiocyanates (I.T.C.s), thiocyanates, sulfates, and nitriles. The last-mentioned compounds give plants a pungent taste and smell and have biological properties, such as antifungal and antibacterial [20,21].

In addition, proteins and peptide fractions, which have a high nutritional profile, have been investigated as attractive molecules in Moringa. These compounds exhibit many biological activities, namely anticancer, antibacterial, antioxidant, hepatoprotective, and antidiabetic. For example, the aromatic and hydrophobic amino acids of the peptides are responsible for the antioxidant activity. Moreover, in *Moringa oleifera* seeds, researchers reported 7 essential and 10 nonessential amino acids, with glutamic acid having the highest value (22.71 g/100 g protein), followed by arginine (15.78 g/100 g protein) [22,23,24].

An underestimated by-product, *Moringa oleifera* seed cake, is obtained after the extraction of the oil, and it has gained attention due to its chemical profile. A higher concentration of Ca and K were observed, and 24 bioactive compounds were identified in the cake residue, such as oleic acid, 3-Hydroxy-2-p-tolyl-2-butenenitril, erucic acid, and eicosanoic acid. Another promising usage of this by-product is to improve the fertility of the soil because of its mineral profile (magnesium, phosphorus, nitrogen copper, calcium, manganese, nickel, zinc, and iron) and the higher quantity of the protein contained (60%) [11,25,26].

Due to the complexity of their chemical composition, all these mentioned bioactive compounds positively impact human health.

### 2.1. Impact on the Human Health

Aside from the high nutritional profile, *M. oleifera* offers many advantages for our health. Different parts of the plant (leaves, seeds, bark, roots, flowers, fruit, and immature pods) have different roles. They can act as an antibacterial, antiasthmatic, diuretic, antidiabetic, antipyretic, anticancer, anti-inflammatory, hepatoprotective, cardiac, and circulatory stimulant [17,27,28,29,30]. A few studies are presented to highlight the benefits of this plant.

Before evaluating the antimicrobial activity against *Pseudomonas aeruginosa* and *Erwinia carotovora*, various parts of Moringa (leaves, flowers, roots, seeds, and bark) were extracted in different solvents, such as methanol, water, acetone, and ethyl acetate. It was found that leaves extracted in methanol, ethyl acetate, and ethanol presented antibacterial action for both mentioned strains [31].

Moreover, Moringa leaves are reported to impact decreasing glucose levels in the blood. This was proved in a study by Momoh (2013), where tablets based on *M. oleifera* leaf powder were orally administered to Wistar albino rats. It was shown that after 8 h, glucose levels were reduced with 54.4%. The tablets also presented good physicochemical properties, and therefore, Moringa leaves can be used as a supplement [32].

In addition, releasing γ-amino butyric acid (GABA) from ethanol extract of *M. oleifera* leaves can treat diseases such as epilepsy by causing anticonvulsing action [33].

Other studied bioactive compounds concerning human health are alkaloids, which are responsible for cardiac stimulation and stabilizing blood pressure. They can also decrease fat and cholesterol by preventing hyperlipidemia [8]. This was proved by Aekthammarat (2019), who studied the effect of an aqueous extract of Moringa leaves on hypertensive Wistar rats. Moringa extract was administered to the Wistar rats at doses of 30 and 60 mg/kg/day, along with N-nitro-L-arginine-methyl ester at a three-week course of 50 mg/kg/day, which increased the animals’ blood pressure and heart rates. After the administration of the extract, blood pressure dropped, and tachycardia occurred [34].

The presence of quercetin released from the alcoholic and aqueous extracts of *M. oleifera* might be responsible for the hepatoprotective activity of the plant [35]. Quercetin, together with other compounds (kaempferol, gallic acid, chlorogenic acid, rosmarinic acid, vicenin-2, and rutin), seem to have another attribution, namely, to heal wounds [36].

Furthermore, it was proven that *Moringa oleifera* had the potential to treat nervous system problems when the Moringa extract, in combination with fluoxetine, was administrated to Swiss albino mice [37]. In addition, the bioactive compounds from the alcoholic fraction of Moringa leaf juice may have antimigraine activity [38].

Another therapeutic potential of *Moringa oleifera* described by the researchers is related to the thyroid. It was shown that a lower dose of Moringa aqueous leaf extract (175 mg kg^−1^ body wt.) is safer and more useful than using a higher dose (350 mg kg^−1^ body wt.) for treating hyperthyroidism [39].

Generally, leaves can treat asthma, hyperglycemia, syphilis, headaches, skin problems, pneumonia, ear infections, and flu. Crohn’s disease, rheumatism, cramps, gout, epilepsy, arthritis, and sexually transmitted diseases might be treated with the Moringa seeds. A remedy for urinary problems is associated with the flowers of Moringa. Moreover, the root bark is used as a cardiac stimulant, and the pods can help with liver, diarrhea, or spleen problems [8,40,41].

The anticancer properties of *M. oleifera* will be described in the following subchapter. It might be considered the most essential activity of the plant because cancer is a prominent cause of death worldwide. Furthermore, some significant anticancer compounds cannot be synthesized chemically due to their complex structures, so they are extracted from different plants [42].

### 2.2. Cytotoxicity of Moringa oleifera

The definitions of cytotoxicity may vary depending on the purpose of the study and whether or not cells are killed or have their metabolism changed. The objective of testing the potential cytotoxicity of the compounds is either because these compounds are believed to be anticancer agents and cytotoxicity may be vital to their effect or because the compounds are used in the pharmaceutical or beauty industry and any harmful side effects must be eliminated [43].

Several bioactive compounds, including alkaloids, flavonoids, and phenolics, have been described in *M. oleifera* as responsible for anticancer and antioxidant activity. It seems that antioxidants inhibit the proliferation of cancer cells. Cell proliferation decreases due to the cytostatic effect, and cell survival decreases due to the cytotoxic effect [44].

To illustrate the cytotoxicity of *M. oleifera* on different cells, some relevant studies are discussed in the following paragraphs.

Starting from 1999, bioactive compounds of *M. oleifera* have been explored. 4 (alpha-L-rhamnosyloxy)-benzyl isothiocyanate, glycerol-1-(9-octadecanoate), 3-O-(6′-O-oleoyl-beta-D-glucopyranosyl)-beta-sitosterol, beta-sitosterol-3-O-beta-D-glucopyranoside, beta-sitosterol, niazimicin, and niazirin, were studied against Epstein–Barr Virus-Early Antigen (EBV-EA). From all the abovementioned, niazimicin seems to be considered a potent antitumor promoter. 4 (alpha-L-rhamnosyloxy)-benzyl isothiocyanate and beta-sitosterol-3-O-beta-D-glucopyranoside also showed significant activity [45].

**Figure 1:**
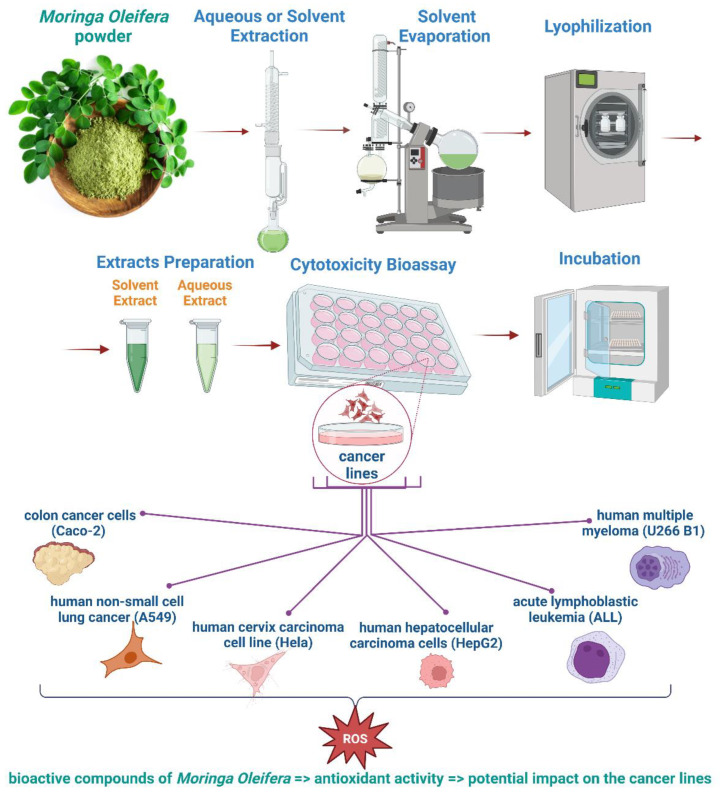
Cytotoxicity bioassay of *M. oleifera* (created with BioRender.com).

The impact of *M. oleifera* Lam. extract against human multiple myeloma (U266 B1) cell lines was investigated by Parvathy and Umamaheshwari. The neutral red dye uptake method was used to test the cytotoxicity of the extract in different solvents, such as methanol, ethanol, ethyl acetate, and chloroform. The results showed that methanolic extract had the most cytotoxic activity in the cells. Some compounds from Moringa leaves similar to vinblastine and vincristine might be used as a herbal treatment for people who suffer from myeloma (i.e., cancer of the plasma cells) [46]. Figure 1 shows an overview of the steps for detecting the cytotoxicity of *M. oleifera.*

The Moringa leaves were encapsulated by spray drying with tragacanth gum as a carrier to increase the bioactivity and bioaccessibility of the compounds. The total polyphenolic content, antioxidant activity, and cytotoxicity (on colon cancer cells—Caco-2) were evaluated on the microencapsulated Moringa. After the in vitro digestion, the total polyphenolic content increased gradually from the mouth (9.7%) to the stomach environment (35.2%) and intestinal one (57.6%). The highest antioxidant activity was associated with the intestinal phase. Regarding cytotoxicity, the Caco-2 cells were tested on microencapsulated and nonencapsulated *M. oleifera* extract. After 48 h of incubation, at the highest concentration (0.125 mg mL^−1^), cell viability was 82% for nonencapsulated and 87% for the encapsulated extract [14].

Moringa leaves extracted in cold water were evaluated for their anticancer activity against human hepatocellular carcinoma cells (HepG2) and human non-small cell lung cancer (A549). When Moringa extracts were orally administered, a significant reduction in proliferation (44–52%) was observed for HepG2 and A549 cells. After these results, the Moringa cold extract might be a treatment for people with liver and lung cancers [47].

Moreover, different extraction methods (cold water, hot water, ethanol 80%) of *M. oleifera* were performed against acute lymphoblastic leukemia (ALL), acute myeloid leukemia (A.M.L.), and HepG2 cells by using the M.T.T. (3-(4, 5-dimethylthiazol-2-yl)-2, 5- diphenyl tetrazolium bromide) assay. After 24 h of incubation, very high activity was observed for the ethanolic extract, with 82% of the A.M.L. cells destructed and 86% of the ALL cells. In contrast, 75% of A.M.L. cells were destroyed by the hot water extract, compared to 84% of ALL cells. Additionally, in the case of HepG2 cells, hot water extract presented the strongest anticancer activity (81% of cells destructed) [42].

Another study was conducted on the human cervix carcinoma cell line (Hela). The *M. oleifera* leaf and callus were extracted in ethanol-water (70–30%), and the test against Hela was carried out using the M.T.T. method. Hela cell viability was considerably reduced by *M. oleifera* callus and leaf extracts; the number of cells survival decreased gradually with increasing concentration. However, *M. oleifera* leaf extract was stronger than the callus extract because of the higher phenolic content. This means that the phenolic compounds are involved in cytotoxicity, which other scientists have also observed [44,48,49].

As we can see from these studies, the bioactive compounds of *M. oleifera* could be used as a natural treatment against different types of cancer due to the prominent source of antioxidants and chemical profile. The cytotoxicity of Moringa seems to be related to its phenolic compounds. Moreover, different extraction methods in cold water, hot water, and solvents might impact the release of the plant’s active ingredients.

## 3. Extraction Techniques Prior to Encapsulation

**Figure 2:**
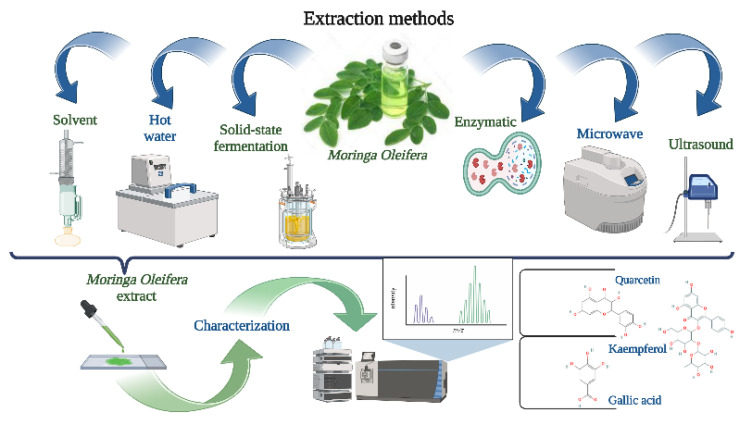
Extraction techniques used before encapsulation of the bioactive compounds from *M. oleifera* (created with BioRender.com).

Over the years, several extraction techniques have been developed (Figure 2), including traditional extraction (solvent, hot water, solid state fermentation) and nontraditional techniques (ultrasound, microwave, enzymatic) [50,51,52,53,54,55,56,57]. Whereas conventional extraction methods include longer extraction durations, toxicity, volatility, flammability, and lower extraction efficiency [58,59,60], nontraditional are considered to be better for the environment since they use fewer synthetic chemicals and produce a better yield and quality of extract [14,61,62,63,64].

### 3.1. Traditional Extraction Techniques

#### 3.1.1. Solvent Extraction

The process of solvent extraction is one of the earliest methods of biological compound extraction. Solvent extraction (solid–liquid or liquid–liquid) requires solvents and heat or stirring. For example, operational factors such as temperature (80–110 °C), solvent ratio (chloroform: methanol from 1:1, 2:1, 3:1, 4:1), and solvent type on the oil extraction yield from Moringa have been examined. Maximum yield (41%) was achieved with increasing temperature (100 °C) and at a solvent ratio of 3:1; however, oil degradation began between 425 and 450 °C [65]. Nobossé et al. investigated how the extraction solvent (methanol, ethanol, and water) and age (30, 45, and 60 days) of fresh Moringa leaves influenced the extraction of the bioactive compounds. Maximum total polyphenols (4.57 g GAE/100 g) and tocopherol content (106 mg/100 g) were all found in leaves aged 60 days by methanol extraction, whereas flavonoids were extracted by ethanol (1.8 g CE/100 g). In addition, total antioxidant capacity was higher in an aqueous solvent. Leaves that were 30 days old had the most significant levels of vitamin C, chlorophyll, carotenoids, and antiperoxide activity (*p* < 0.05) [66].

However, in order to use the extract in food applications, it is still necessary to identify the optimum extraction solvents and to determine if extraction generates cytotoxicity [67,68]. It has been shown that methanolic solvents used to extract biological compounds from *M. oleifera* leaves had 17 times higher antioxidant activity than aqueous solvents (Trolox equivalents of 160.18 nmol/mL). The aqueous extract was less cytotoxic than the methanolic, as cells that resulted were more susceptible to concentrations ranging from 0.05 to 5 mg/L. [68]. In addition, the ethanolic extract of *M. oleifera* seeds at 1000 mg/kg had a significant decrease in erythrocytes in Wistar rats [67]. Despite the drawbacks (toxicity, volatility, flammability), traditional solvent techniques are still commonly used since the extraction units are easily accessible and less expensive than nontraditional methods.

#### 3.1.2. Hot Water

Since the selective extraction process, the route from Moringa to bioactive recovery is challenging [51,52,54,58]. It has been reported that temperature is the primary factor that impacts the selectivity and recovery of phenolic compounds [51,52,58]. To fully extract the phenolic and flavonoid components, several researchers have also suggested that the maximum temperature for hot water extraction should be lower than 200 °C [51,54,58]. For example, the effect of temperature (50–200 °C) and duration (5–60 min) on the extractability pattern of macronutrient (Ca, K, and Mg) and micronutrient components (Al, Co, Cr, Cu, Fe, Ni, and Zn) from *M. oleifera* leaves during hot water extraction was evaluated. The proper temperature and extraction time were discovered to be 90 °C and 60 min, respectively. Micronutrient extraction improves with temperatures over 90 °C, which may increase the risk of recovering toxic elements from the extract [51].

In another study, at different extraction temperature (25–200 °C), the behavior of three flavonols’ (myricetin, quercetin, and kaempferol) content of Moringa leaves powder was characterized. Myricetin (2699 mg/kg) and kaempferol (3440 mg/kg) concentrations were found to be at their highest at 100 °C, but they began to decrease (1496 mg/kg, and 1730 mg/kg, respectively) at 150 °C. However, the concentration of quercetin (1429–1480 mg/kg) remained constant as the extraction temperature increased [58].

Comparing pressurized hot water extractions and carbon dioxide-expanded ethanol, the maximum yield of total phenolics from Moringa leaves was obtained for pressurized hot water extractions (62.4 vs. 20.3 mg GAE/g leaves) [54].

#### 3.1.3. Solid-State Fermentation

Today’s world continuously develops, and creativity is the fundamental impetus for technical progress and innovation. For this reason, researchers are trying to find more sustainable methods, such as solid-state fermentation (S.S.F.). It is a fermentation method with a solid substrate/support and flow-free water. To overcome the low organoleptic properties and digestibility of Moringa leaf, extracts from leaf flour were investigated following S.S.F. with *Aspergillus niger*. The fermentation process was performed at 30 °C for 168 h using spore suspension (1.0–4.0 × 10^7^/g of solid) and different concentrations of extraction solvents (40% ethanol (*v/v*), 40% acetone (*v/v*), 80% ethanol (*v/v*), and 80% acetone (*v/v*)). The highest levels of total phenolics (136.4%) and flavonoids (783.1%) were identified using extraction solvents (80% ethanol and 80% acetone, respectively). Moreover, in vitro antioxidant activity in ferric reduction (277.2 mol Trolox/g M.L.F.) was observed with the 80% acetone solvent [59]. Zhang et al. used four bacterial strains (*Trichoderma reesei* CICC 2626, *Bacillus pumilus* CICC 10440, *Bacillus subtilis* subsp. CICC 21934, and *Bacillus* sp. CICC 21942) for S.S.F. The most soluble protein from Moringa leaf powder (285 mg/g) was generated utilizing *Bacillus pumilus* CICC 10440 in a 1:60 ratio over 24 h [60]. S.S.F. is a dynamic replacement for renowned traditional methods of metabolite processing that plays a significant role in producing food or biofuels.

### 3.2. Nontraditional Extraction Techniques

#### 3.2.1. Ultrasound

Ultrasound extraction (U.E.) is based on cavitation, which disintegrates cell walls and releases active compounds from their natural matrices [69]. The cavitation that contains the active compounds will be compressed during sonication, providing a high-temperature and pressure microenvironment, thus speeding up the extraction process [56]. One study used response surface methodology to determine the optimum conditions for performing U.E. for *M. oleifera* leaves with methanol extraction solvent. Most active compounds (quercetin 3-glucoside, quercetin malonyl glucoside) were released at 11 °C, pH 7.7, time of 15 min, and a methanol concentration of 74.5% [56]. Pollini et al. highlight the impact of solvent composition in the U.E. on the biological properties of *M. Oleifera* leaves. The highest concentrations (216.4 µg/g quercetin 3-O-rhamnoside and 293.9 µg/g quercetin 3-O-(6″-O-malonyl)-β-D-glucoside) were found under the following U.E. factors: 50% water, 60:1 liquid/solid ratio, and 60 °C for 60 min. [53]. In addition, the effectiveness of the U.E. in increasing the extraction efficiency of oil from *M. oleifera* seeds is also studied by several researchers [50,57,70]. There were no significant differences between U.E. and conventional extraction in oil yield, but the time for oil extraction, U.E. (20 min), and conventional extraction (50 min) was significantly lower (*p* < 0.05) [57].

#### 3.2.2. Microwave

Due to the polar solvents’ dipolar rotation, microwave radiation can cross the material structure, causing molecular friction that speeds up the mass transfer of the molecules of interest [14]. As reported by Chen et al. and Da Porto et al., microwave extraction (M.E.) may be used to preheat seeds before oil extraction [14,62]. The extracted oil using the Soxhlet method was more successful when M.E. pretreatments were performed (40.0%) at 100 W for 30, 60, and 90 s compared to the extracted oil using untreated seeds (+9.9%) [62].

Based on their research, Rodriguez et al. suggest the following M.E. optimal parameters for Moringa leaves: 20 min of extraction time, 42% ethanol, and 158 °C. The extraction yields on 26 ± 2%, 15 ± 2 g/mL DPPH, Trolox equivalent antioxidant capacity 16 ± 1 Trolox/100 g dry leaf [55]. Moreover, for the protein extraction, ME conditions (solvent–solid ratio 91:1 mL/g, extraction time 148 s, extraction temperature 41°C, microwave power 81 W) showed significantly high extraction efficacy (82.07 mg/g) compared to conventional solvent extraction method (68.99 mg/g) [61].

#### 3.2.3. Enzymatic

Enzyme extraction (E.E.) seems to be a promising advanced technique for selecting biological compounds compared to the traditional solvent process [63,64]. For example, Latif et al. [63] use a commercial enzyme (protease; 580 000 DU/g) to extract oil and protein from the Moringa seed. The weight percentage of oil extracted by E.E. (22.5%) was significantly higher than that of aqueous extraction (7.8%) but significantly lower (*p* < 0.05) than hexane extraction (32.4%). Regarding protein content in the aqueous and creamy phase, E.E. shows the highest range (25.8%) compared to aqueous extraction (15.0%). Moreover, it was suggested that by using E.E. (protease and cellulase), the maximum oil recovery (70%) was achieved with a pH of 4.5, a moisture/kernel ratio of 8:1 (*w*/*w*), and a shaking rate of 300 strokes/min at 40 °C for a 1 h incubation period [64]. For the E.E. leaf protein, pH and the enzyme/substrate ratio had no significant impact, whereas the essential factors, including temperature 30 °C, multienzyme complex concentration 60 fungal beta-glucanase units (arabinose, cellulase, ß-glucanase, hemicellulose, and xylanase enzyme), and incubation time of 0.3 h have a substantial influence [71].

Enzymatic treatment can potentially improve polyphenol extraction yield and reduce energy consumption with lower process temperatures compared to current methods.

## 4. Encapsulation Methods of Bioactive Compounds from *Moringa oleifera*

Since the bioactive molecules extracted from different parts of *M. oleifera*, in most cases, cannot be used in food applications as they are, encapsulation is proposed. For example, *M. oleifera* leaf extracts have low bioavailability, limited water solubility, and are very unstable and oxidation-prone [13]. *M. oleifera* bioactive compounds react to several elements used in food processing procedures (pH, water activity, light conditions, oxygen, and temperature). Therefore, it is important to stop them from deteriorating and increase their stability in those circumstances. Encapsulation technology can offer a strong physical barrier against the abovementioned elements [14].

**Figure 3:**
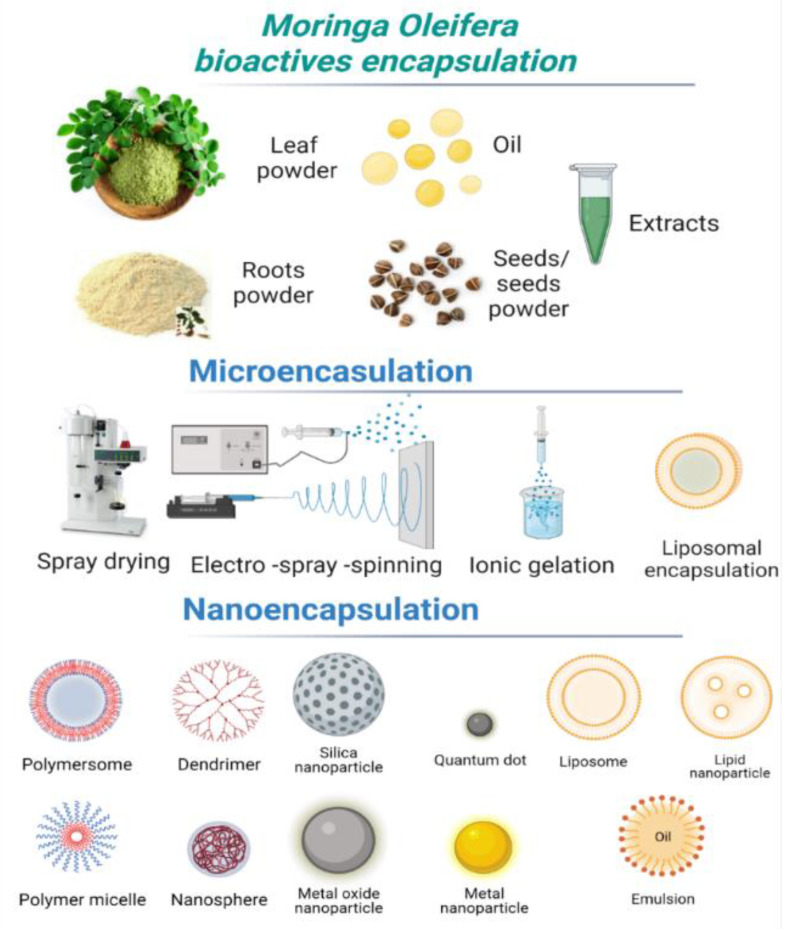
Most-studied encapsulation methods for *M. oleifera* bioactive compounds (created with BioRender.com).

To create microcapsules that have various desirable qualities, the essential bioactive chemicals are protected by encapsulation in either a homogeneous or heterogeneous matrix. By using this method, unstable liquid compounds can be converted into free-flowing stable powder. Core bioactive components have been encapsulated using a variety of techniques, including cross-linking gelation, freeze-drying, microemulsion, spray drying, and different nanoformulations (nanoliposomes) (Figure 3) [72,73,74].

Microencapsulation makes the bioactive compounds easier to handle while protecting them from light, air, and other elements. In the encapsulation process, biopolymers (alginate, chitosan, etc.) and polysaccharides (maltodextrin, starch, etc.) are frequently used as carriers or coatings along with animal-based (whey) or plant-based proteins (soy, pea proteins) and gums (arabic, acacia gum) [13,64,65,66,67]. The optimal encapsulation material should have excellent water solubility, good emulsification, and film-forming properties, quick drying rate, and low viscosity, even in a highly concentrated solution [75]. Of the encapsulating techniques, spray drying is the least expensive and most widely used for turning emulsions into stable powder [64] with increased bioavailability, reduced oxidation rate, controlled release, and the unpleasant flavor masked.

Encapsulation parameters, such as nozzle size, inlet temperature, flow rate, outlet temperature, rotations per minute, surfactant, polymer solution feed rate, and cross-linking agent concentration directly influence the yield of the process, the size and the shape of the obtained particles, and the amount of bioactive compound [13]. For example, Airouyuwa (2019) observed that the yield of the spray drying was higher at the highest temperature (140, 160, and 180 °C), explained by the fact that when using greater inner temperature, the capacity of the heat and mass transfer process are increased and the evaporation rate is higher [13]. In obtaining microemulsions, the time, temperature, and rotations influence the emulsion and the bioactive compound stability. In this process, the type of oil phase used and the amount of bioactive material impact the microemulsion size and appearance (for a clear, transparent, or translucent microemulsion, the droplet sizes must be under 100 nm) [76,77]. The distance between the pipette nozzle and the surface of the hardening bath during the cross-linking gelation is a factor that directly affects the size and shape of the beads.

Additionally, it has been shown that phenolic compounds can reduce the surface tension of solutions, which might account for the observed reduction in particle size [78]. Interestingly, in the electrospraying process, surface tension repulsion outweighs viscoelastic forces at relatively low concentrations of gelatin (7%), causing beads to form. However, if core concentration rises and a mixed bead-fiber structure develops, this equilibrium may be harmed [79]. Among the other already-stated parameters, the polymer concentration, molecular weight, and ratio between polymers (when a mixture) can modify the shape and size of the obtained particles [78,80]. As a result of the improvement in surface-to-volume ratio, encapsulated materials with smaller particle sizes are more advantageous for food applications. Overall, these results suggest that well-designed and tailored encapsulation protocols can maximize the use of *M. oleifera* bioactive compounds.

### 4.1. Microencapsulation of Moringa oleifera and Their Food Applications

Due to the nature of its components, *M. oleifera* has many applications. The seed powder is utilized as a natural coagulant for water treatment, removing many suspended particles from rivers and murky streams [81]. The oil from the seeds is used for cosmetics, biodiesel production, and even as a fertilizer. Zeatin may be made from *M. oleifera* extracts, which promote plant growth and raises crop yields [82]. In addition to these uses, *M. oleifera* utilization in different food matrixes is becoming more widespread. For instance, in Mexico, fishmeal was replaced with *M. oleifera* due to the plant’s high protein and carbohydrate content in tilapia feed [83]. Oyenyinca (2018) and his collaborators describe how different bioactive compounds belonging to *M. oleifera* are used in various foods as fortificants, mostly in an encapsulated form.

However, they conclude that it might not always be considered fortification or enrichment when *M. oleifera* is used to enhance the nutritional content of staple foods [84]. It would seem from the results presented by Dollah (2014) that mixing *M. oleifera* oil with other vegetable oils would make it possible to modify or alter the original properties of the oils and provide functional and nutritional attributes for use in various food applications, expanding the opportunities for the commercial use of these oils [85].

To extend the shelf life of chicken burgers, Alqurashi et al. investigated the antibacterial properties of *M. oleifera* leaves extract (0.5–2%). The results showed that chicken burgers with 1–2% extract had a significant impact (*p* < 0.05) in reducing the total number of plates up to 6 days of refrigeration storage at 4 °C [86]. Moreover, through cross-linking gelation method, the addition of alginate encapsulation of *Lactobacillus plantarum* (9.32–9.58 Log10 CFU/g) and *Pediococcus acidilactici* (9.82–9.81 Log10 CFU/g) with *Moringa oleifera* extract (0.05–0.1%) in fruit juice and drinkable yogurt was evaluated. After 28 days of storage, the plant extract increased bacterial stability compared with the control in both products [87]. Table 1 shows the most-used encapsulation techniques for different bioactive compounds from *M. oleifera* and their possible applications. Nevertheless, despite their health advantages, additional human/clinical trials and evaluations are required to further rule out any health concerns [5,11].

### 4.2. Nanoencapsulation

Recently, nanoencapsulation has been used as a strategy for overcoming the drawbacks of traditional drug delivery methods, such as low bioavailability, adverse reactions, and unpleasant organoleptic qualities [79,91,92,93,94].

The effectiveness of nanoencapsulation ethanolic *M. oleifera* leaf extract in a fish-sourced gelatin matrix was assessed. *M. oleifera* extract encapsulation at 1%, 3%, and 5% produced bead-free and uniform nanofibers. According to scanning electron microscopy (S.E.M.) and transmission electron microscopy (T.E.M.) analysis, pure gelatin and encapsulated nanofiber have internal dimensions of 115 nm and 39 nm, respectively. Encapsulation efficiency was 80–85%, and toxicological results suggest that the extract was safe for consumption [79]. Moreover, a symbiotic containing *Saccharomyces cerevisiae* yeast (11 × 10^12^ CFU) and *M. oleifera* leaf extract was developed for growing rabbits using the nanoencapsulation technique. Alginate (1% *w/v*) and CaCl_2_ (0.5% *w/v*) successfully encapsulated the supplement (51.38 nm) with encapsulation efficiencies of 57.55% [91].

For the biocontrol of *Listeria monocytogenes* and *Staphylococcus aureus* (10^3^ CFU/g) on cheese, an active food packaging material with Moringa oil (20 mg/mL)-loaded chitosan (3.0 mg/mL) nanoparticles (MO@CNPs) and MO@CNP (9.0 mg/mL)-embedded gelatin nanofibers has been developed. When applied to cheese, MO@CNPs nanofibers showed solid antibacterial activity (78.63% and 98.67%, respectively) at 4 °C for 10 days, extending the shelf life with no negative Impact on the flavor or texture of the cheese [92]. Moreover, alginate nanoparticles (0.1%) with *Moringa oleifera* extract (0.1%) showed antimicrobial activity against *Staphylococcus aureus*, *Escherichia coli*, *Proteus vulgaris*, *Citrobacter diversus*, and *Salmonella enterica* [95].

Nanoencapsulation is still one of the most promising technologies because of its potential to encapsulate bioactive components.

### 4.3. Electrospinning Method

Electrospinning has been used to make active food packaging with incorporated antioxidants, antiripening, and antimicrobials to maintain fresh and processed foods safe and increase their shelf life [96,97]. Particularly, nanofiber products made by electrospinning have received a lot of attention due to their better filtration efficiency and breathability. For example, different polymer concentrations (30–45%) and an injection flow rate (0.7 mL/h) of zein nanofiber with *M. oleifera* kernel oil were evaluated. Hydrophobic electrospun nanofibers averaged 450 ± 24 nm and encapsulated the oil efficiently, thus prolonging the oil shelf life [97]. Narayan et al. [93] show the beneficial effect (∼88%) of 7% polyacrylonitrile-supported biosorbent (*M. oleifera* 20 mg/mL) for the absorption of Congo red dye from an aqueous solution.

More research into electrospinning technology to generate biological macromolecule materials for the encapsulation and sustained release of food-active compounds is required.

### 4.4. Electrospraying Method

Electrospraying has been proposed to produce biological microcapsules; however, its usage in food encapsulation is currently unexplored [94,98]. For the encapsulation of the bioactive extract from *M. oleifera* (1–5%, *w*/*w*) through the electrospraying technique, a gelatin matrix (7.5%) was used to produce nanocapsules (140–179 nm). Electrospraying parameters were 20 kV voltage, 0.5 mL/h flow rate, and 10 cm emitter/collector distance; thus, extract-loaded nanocapsules had an encapsulation effectiveness of 83.0 ± 4.0% [79]. Additionally, the physical and antioxidant characteristics of chocolate alginate beads with *Moringa oleifera* leaf extract (0–6%) were assessed. Extract concentration (6%) made the beads smaller (4.3 mm) and more spherical, whereas hardening time just influenced circularity. For beads prepared with 2% extract and allowed 20 min in CaCl_2_, a minimum total phenolic concentration of 0.84 mg GAE/g beads was reported. However, the radical scavenging activity was concentration-related and required a level of 6% MLE to obtain inhibitory impact [78].

According to these studies, electrospraying is a more feasible and efficient alternative to encapsulate biological compounds than spray drying, which uses high temperatures [94,98].

## 5. Conclusions and Future Directions

A variety of therapeutic properties (anticancer, antibacterial, antiproliferative, antihypertensive, and anti-inflammatory) of bioactive compounds belonging to *M. oleifera* leaves, roots, seeds, and oils are sustained by in vitro and in vivo studies.

Including *M. oleifera* Lam. bioactive molecules in the humans diet are crucial for improving food nutrient value, human health sustainment, and environmental and sustainability reasons. The impact of these bioactive molecules on their applications is dependent primarily on two characteristics: on the one hand, their activity and, on the other hand, stability. These traits are closely related to their structural elements and ability to incorporate and maintain their bioactive features in the food matrix.

A combination of conventional and cutting-edge methodologies, such as thermal, chemical, and biotechnological methods, can be used to increase their use in food and beverages. Encapsulation is one of the advanced methods that has recently gained interest in the food sector. The bioactive compounds of *M. oleifera* can be encapsulated for optimal delivery within the human stomach or intestinal tract to produce pleasant, safe, and affordable foods. Therefore, compared to everyday food items, highly nutritious foods rich in *M. oleifera* bioactive compounds will need to be customized more precisely. In addition, using this valuable technique must ensure cost efficiency and sustainability. After analyzing the literature, we can conclude that spray drying is a cost-effective method, and it is very versatile. As we have seen, encapsulation efficiency can be maximized by using specific biopolymers, especially proteins or protein derivatives. The entrapped compounds’ stability also supports the use of this technique over time. Studies related to the sustainability of this method should be started, considering that obtaining the encapsulation matrices must also be part of this objective.

However, these encapsulations can overlap concerns regarding the cytotoxicity of the *M. oleifera* compounds. In addition to studying the cytotoxicity of Moringa bioactive compounds, the cytotoxicity of the carrier materials has also become an interesting topic for researchers. However, more studies in this direction have to be explored. Furthermore, we believe that implementing sustainable extraction methods and well-designed encapsulation techniques can maximize the utilization of *M. oleifera* bioactive compounds in the food industry, cosmetics, pharmaceutics, and water cleaning. More precisely, using green extraction methods, biopolymers, adequate concentrations, and suitable encapsulation methods will increase the benefits derived from the use of *M. oleifera* bioactive compounds. Because new encapsulation techniques involve pricy devices, we concluded that spray drying is the most suitable technique for designing and implementing the bioactive compound’s application in human health, with good yield, a broad palette of applicability, and high versatility.

## Figures and Tables

**Table 1:** *Moringa oleifera* bioactive compounds encapsulation techniques and features of the encapsulated product.

Encapsulation Technique	Encapsulated Material	Encapsulation Matrix	Encapsulation Formulation	Features	Application	Ref.
Cross-linking gelation	*Moringa* *oleifera* leaf extract	Alginate, whey protein, cacao powder	0, 2, 4, and 6% extract concentration	♦ beads size 4.30–5.02 mm ♦ phenolic content 0.88–1.38 mg GAE/g of beads ♦ 20–50 mL/L dissolved ♦ organic carbon release in the first 30 min	food ingredient	[78]
	*Moringa* *oleifera* seed powder (MSP)	Sodium alginate	♦ 3% Alg, 2% MSP ♦ 2% Alg, 1% MSP ♦ blank 2%/3% Alg	♦ ↑perfluoroalkyl and polyfluoroalkyl substances absorption ♦ 90–95% bead moisture content	pollutants removal	[73]
	*Moringa* *oleifera seed* powder (MSP)	Sodium alginate		♦ mean diameter 3 mm ♦ Cu^2+^ 99.3%, Ni^2+^ 77.98%, Co^2+^ 70.80%, Zn^2+^ 60.90%, Mn^2+^ 49.96% biosorption	pollutants removal	[81]
Freeze-drying	*Moringa* *oleifera* leaf powder ethanol extract	Maltodextrin, arabic gum	Malto-dextrin, arabic gum	♦ moisture content 1.47–1.77% ♦ hygroscopicity 11.13–15.86% ♦ water solubility index 86.35–98.74%	food fortification	[88]
Micro-emulsion	Carotenoids *Moringa* *oleifera* L. leaves	Lecithin, sunflower oil, absolute ethanol, water		♦ encapsulation efficiency 59.67% ♦ stable 24 h at room temperature ♦ stable after centrifugation ♦ stable under thermal test	antioxidant	[76]
	*Moringa* *oleifera* leaves extract	Coconut/ soybean oil, tween80, span80	4%, 8% *Moringa* *oleifera* leaves extract	♦ microemulsion size 15.1–82 nm ♦ stable after centrifugation ♦ stable under thermal test	food fortification	[77]
Spray drying	Drumstick oil (30%)	Maltodextrin (MD) Gum Arabic (GA) Whey Protein (WP)	MD: GA (25:75, 50:50, 75:25) MD: WPC (25:75, 50:50, 75:25)	♦ encapsulation efficiency 66.23–91.05% ♦ surface oil content 1.65–6.11% ♦ moisture content 1.44–1.91% ♦ particle size distribution 11.43–28.03 µm ♦ density 1.15–1.34 g/cm^3^ ♦ porosity 0.36–0.58 ♦ ↑ oil stability	food fortification	[89]
	Polyphenols (*M. oleifera*) extracted by microwave- assisted extraction	Tragacanth gum (TG), locust bean gum (LBG), and carboxymethyl-cellulose (CMC)	TG 1%, LBG 1%, CMC 1%, LBG 0.5% + TG 0.5%, TG 0.5% + CMC 0.5%, CMC 0.5% + LBG 0.5%	♦ encapsulation efficiency 24.28–35.74% ♦ total phenolic content 25.17–27.49 (mg GAE g^−1^ of microparticles) ♦ total flavonoid content 23.20–26.82 (mg QE g^−1^ of microparticles) ♦ DPPH 5.96–6.95 (mg GAE g^−1^ of microparticles) ♦ ABTS 5.61–6.18 (mg TE g^−1^ of microparticles)	antioxidant	[74]
	*Moringa* *oleifera* leaves extract (MLE)	Soy protein isolate (SPI), pea protein isolate (PPI)	10% plant-based protein 11–22% (*w/v*) (MLE)	♦ encapsulation yield 65.46–85.22% (SPI)/63.63–88.64% (PPI) ♦ total polyphenols content 6.76–11.96 mg GAE/g powder (SPI formulation) and 4.95–8.81 mg GAE/g (PPI formulation)	antioxidant	[13]
	*Moringa* *oleifera* leaves extract (MLE)	Tragacanth gum	1% tragacanth gum	♦ total polyphenols 42.47–45.28% (from the initial content) ♦ antioxidant activity 39.7–75.32% across 30 days ♦ storage ♦ polyphenols release in simulated gastrointestinal test 9.7% oral conditions, 27.2–35.2% gastric conditions (30–60 min), 44.1, 51.8 and 57.6% intestinal conditions (2, 3 and 4 h)	antioxidant	[90]

## Data Availability

Not applicable.
